# Potential Protein Signatures for Recurrence Prediction of Ischemic Stroke

**DOI:** 10.1161/JAHA.123.032840

**Published:** 2024-02-29

**Authors:** Chengyi Zhang, Yang Liu, Huimin Zhu, Xinying Huang, Cang Guo, Si Cheng, Meng Yuan, Yong Jiang, Xia Meng, S. Claiborne Johnston, Yongjun Wang, Wei‐Na Jin, Fu‐Dong Shi

**Affiliations:** ^1^ Center for Neurological Diseases, China National Clinical Research Center for Neurological Diseases, Beijing Tiantan Hospital Capital Medical University Beijing China; ^2^ School of Population Medicine and Public Health Chinese Academy of Medical Sciences & Peking Union Medical College Beijing China; ^3^ Changping Laboratory Beijing China; ^4^ Dell Medical School University of Texas at Austin Austin TX USA

**Keywords:** plasma, proteomics, stroke recurrence, Biomarkers, Ischemia

## Abstract

**Background:**

Acute ischemic stroke is a major cause of mortality and disability worldwide, with approximately 7.4% to 7.7% recurrence within the first 3 months. This study aimed to identify potential biomarkers for predicting stroke recurrence.

**Methods and Results:**

We conducted a nested case–control study using a hospital‐based cohort from the Third China National Stroke Registry selecting 214 age‐ and sex‐matched patients with ischemic stroke with hypertension and no history of diabetes or heart disease. Using data‐independent acquisition for discovery and multiple reaction monitoring for quantitative validation, we identified 26 differentially expressed proteins in large‐artery atherosclerosis (Causative Classification of Ischemic Stroke [CCS]1), 16 in small‐artery occlusion (CCS3), and 25 in undetermined causes (CCS5) among patients with recurrent stroke. In the CCS1 and CCS3 subgroups, differentially expressed proteins were associated with platelet aggregation, neuronal death/cerebroprotection, and immune response, whereas differentially expressed proteins in the CCS5 subgroup were linked to altered metabolic functions. Validated recurrence predictors included proteins associated with neutrophil activity and vascular inflammation (TAGLN2 [transgelin 2], ITGAM [integrin subunit α M]/TAGLN2 ratio, ITGAM/MYL9 [myosin light chain 9] ratio, TAGLN2/RSU1 [Ras suppressor protein 1] ratio) in the CCS3 subgroup and proteins associated with endothelial plasticity and blood–brain barrier integrity (ITGAM/MYL9 ratio and COL1A2 [collagen type I α 2 chain]/MYL9 ratio) in the CCS3 and CCS5 subgroups, respectively.

**Conclusions:**

These findings provide a foundation for developing a blood‐based biomarker panel, using causative classifications, which may be used in routine clinical practice to predict stroke recurrence.

**Nonstandard Abbreviations and Acronyms**
AIS: acute ischemic stroke
AK: adenylate kinase isoenzyme 1
ARL6IP5: ADP ribosylation factor like GTPase 6 interacting protein 5
BID: BH3‐interacting domain death agonist
CA3: carbonic anhydrase 3
CCS: Causative Classification of Ischemic Stroke
CNSR‐III: Third China National Stroke Registry
CO1A2: collagen type I α 2 chain
DBNL: Drebrin‐like protein
DEP: differently expressed protein
ENO1: α‐enolase
GNAQ: G protein subunit α Q
GSTP1: glutathione S‐transferase Pi 1
HRNR: hornerin
ITGAM: integrin subunit α M
MRM: multiple reaction monitoring
MYL9: myosin light chain 9
PARK7: protein/nucleic acid deglycase DJ‐1
PRDX1: peroxiredoxin‐1
RSU1: Ras suppressor protein 1
SPTB: spectrin β
TAGLN2: transgelin 2
TREML1: Trem‐like transcript 1 protein

**Clinical Perspective**

**What Is New?**

- This is a proteomic study to identify a biomarker panel to predict stroke recurrence within Causative Classification of Ischemic Stroke subtypes.
- The function of differential expressed proteins detected in this study points the way to future stroke recurrence prevention strategies.

**What Are the Clinical Implications?**

- This study has provided 15 protein panels that could predict stroke recurrence in different Causative Classification of Ischemic Stroke subtypes.

Acute ischemic stroke (AIS) is a leading cause of mortality and disability worldwide. According to the Global Burden of Disease study in 2019, the age‐standardized incidence of stroke ranged from 81.9 to 110.76 per 100 000 person‐years. Stroke accounted for 143 million disability‐adjusted life‐years and 6.55 million deaths.^1^ During the initial 3 months after stroke, patients experience a recurrence rate of approximately 7.4% to 7.7%.^2^, ^3^ The risk of first recurrent stroke is 6 times higher than that of a first‐ever stroke in the general population of the same age and sex.^4^ Furthermore, the 30‐day case fatality rate after the first recurrent stroke is significantly higher at 41% compared with 22% after a first‐ever stroke.^4^ Identifying reliable biomarkers for predicting stroke recurrence is crucial for tailoring effective preventive strategies and improving patient outcomes.

Clinical characteristics, such as poststroke cognitive impairment, heavy smoking, white matter hyperintensity load, biological age, and socioeconomic status, have been recognized as factors influencing stroke recurrence.^5^, ^6^, ^7^, ^8^ The Causative Classification of Ischemic Stroke (CCS) system demonstrates that the risk of 90‐day recurrence was 10.9% in large‐artery atherosclerosis (CCS1), 0.9% in small‐artery occlusion (CCS3), and 14.2% in undetermined causes (CCS5).^9^ However, the heterogeneity of symptomatic phenotypes in studied patients makes it challenging to determine the mechanism linking these features to stroke recurrence. Moreover, the predictive algorithms that use clinical and radiological parameters to forecast stroke recurrence often exhibit a lack of calibration and discrimination. Thus, gaining a better understanding of the underlying causes of recurrent stroke is essential for developing a stratified approach to clinical management.^10^

Circulating blood contains proteins that can indicate abnormalities in the brain or vascular system.^11^ Proteomics, an emerging field, offers a powerful tool for investigating the complex molecular mechanisms underlying various diseases, including ischemic stroke.^12^ Through the analysis of unique protein expression patterns in biological samples, proteomics has the potential to uncover novel biomarkers that can aid in early detection, risk stratification, and therapeutic decision‐making. In this study, using a hospital‐based cohort from the CNSR‐III (Third China National Stroke Registry), we conducted a high‐throughput, nontargeted plasma proteomics analysis to identify potential biomarkers associated with stroke recurrence in the CSS subtypes. The findings from this study have the potential to enhance our understanding of cerebrovascular pathologies and translate into clinical management of stroke.^13^

## Methods

### Data Availability

All data for this study are available from the corresponding author upon reasonable request.

### Research Population Analysis and Sample Collection

This is a substudy of the CNSR‐III, a prospective, multicenter, observational study conducted across 201 hospitals in China from August 2015 to March 2018. The medical ethics committee of Tiantan Hospital approved this study (KY2015‐001‐01), and informed consent was obtained from each participant or their representatives. The CNSR‐III included patients aged ≥18 years, diagnosed with AIS or transient ischemic attack within 7 days from symptoms onset to enrollment. Patients with silent cerebral infarction without signs and symptoms were excluded. Further details about the CNSR‐III study design can be found in the previously published study protocol.^14^

**Figure 1:**
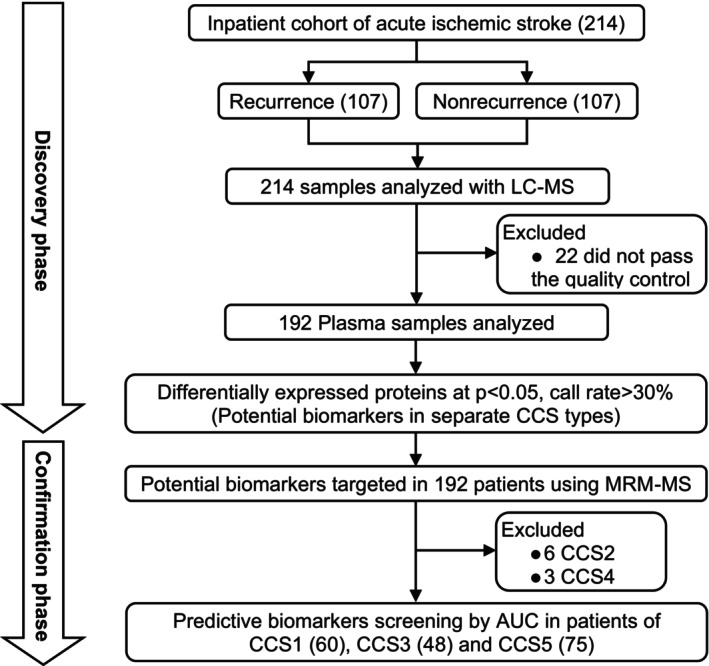
Study design for patients' enrollment and the samples collection for protein discovery and validation. AUC indicates area under the curve; CCS, Causative Classification of Ischemic Stroke; LC‐MS, liquid chromatography tandem mass spectrometry; and MRM‐MS, multiple reaction monitoring mass spectrometry.

In this proteomics study, we focused on patients with a history of hypertension and no history of diabetes or heart disease, selecting them from the 15 166 patients in the CNSR‐III study. AIS recurrence was defined according to World Health Organization criteria, involving the occurrence of a new acute neurologic deficit lasting >24 hours and not attributable to other causes of neurologic deterioration confirmed by magnetic resonance imaging or brain computed tomography scan. All patients underwent a face‐to‐face interview at the 3‐month follow‐up visit. The patient selection and proteomics analysis protocol are summarized in Figure 1.

The CCS system demonstrates greater prognostic value for events such as 90‐day recurrence or death compared with other classification systems.^9^ To ensure compatibility, patients without stroke recurrence were matched 1:1 with patients who experienced stroke recurrence based on sex and age. We mainly focused on 3 CCS subtypes, including CCS1, CCS3, and CCS5. Clinical information was collected using a standardized data collection protocol, including modified Rankin Scale, National Institutes of Health Stroke Scale score, age, blood pressure, clinical features, biological sample (blood), and imaging data.^14^

Peripheral blood was collected within 7 days after the first acute ischemic stroke attack in EDTA tubes (BD Vacutainer, Franklin Lakes, NJ) and centrifuged within 30 minutes of collection at 900*g* for 10 minutes at 2 °C to 8 °C. The plasma was then divided into aliquots and stored at −80 °C until proteomics analysis was conducted.

### Protein Extraction and Digestion

Cellular debris was eliminated from plasma samples through centrifugation at 12 000*g* for 10 minutes at 4 °C. Subsequently, the supernatant was transferred to a new centrifuge tube. The Pierce Top 14 Abundant Protein Depletion Spin Columns Kit (ThermoFisher Scientific) was used to remove the 14 proteins with the highest abundance. Finally, the protein concentration was determined using the BCA kit according to manufacturer's instructions.

For digestion, the protein solution was supplemented with 8 M urea and reduced with 5 mM dithiothreitol for 30 minutes at 56 °C. Following that, the protein solution was alkylated with 11 mM iodoacetamide for 15 minutes at room temperature in the dark. Next, the protein sample was diluted by adding 100 mM triethylammonium bicarbonate buffer to achieve a urea concentration of <2 M. Trypsin was added at a trypsin‐to‐protein mass ratio of 1:50 for the initial overnight digestion and at a ratio of 1:100 for the subsequent 4‐hour digestion. Finally, the peptides were desalted using a C18 solid phase extraction cartridge.

### Liquid Chromatography Tandem Mass Spectrometry Analysis

During the discovery phase, next‐generation label‐free quantitative proteomics technology was used to analyze plasma samples. The data‐independent acquisition mode was used, and the samples were normalized to total protein content. Tryptic peptides were dissolved in solvent A (0.1% formic acid, 2% acetonitrile in water) and directly loaded onto a homemade reverse‐phase analytical column (25 cm long, 75/100 μm ID). Peptides were separated on a gradient of 6% to 24% solvent B (0.1% formic acid in acetonitrile) over 70 minutes, then on a gradient of 24% to 35% solvent B over 14 minutes, and climbing to 80% solvent B over 3 minutes, then holding at 80% for the last 3 minutes, all at a constant flow rate of 450 nL/min using a nanoElute ultra high performance liquid chromatography system (Bruker Daltonics).

The peptides were subjected to capillary source followed by timsTOF Pro (Bruker Daltonics) mass spectrometry, with an electrospray voltage of 1.60 kV. Precursors and fragments were analyzed at the time‐of‐flight detector, with a MS/MS scan range from 100 to 1700 m/z. The timsTOF Pro operated in parallel accumulation serial fragmentation mode. Precursors with charge states ranging from 0 to 5 were selected for fragmentation, and 10 parallel accumulation serial fragmentation mode–MS/MS scans were acquired per cycle. The dynamic exclusion was set to 30 seconds.

### Proteomics Data Processing

The MS/MS data were processed using the MaxQuant search engine (v.1.6.15.0). Tandem mass spectra were matched against the human SwissProt database, consisting of 20 422 entries, and concatenated with a reverse decoy database. The false discovery rate was adjusted to be <1%. Protein expression values were normalized to ensure equal sums across each sample. Among the 1788 detected proteins, only 1084 proteins with a call rate >30% in both the stroke recurrence and stroke nonrecurrence groups were retained. All protein expression values were subjected to log2‐transformation.

Significantly differently expressed proteins (DEPs) were defined as proteins with a fold change >1.5 or <0.67 from the raw value, accompanied by a *P* value of <0.05 determined by Student *t* test. Differential expression analyses were conducted separately for the CCS1, CCS3, and CCS5 subtypes, and the resulting DEPs were used for Gene Ontology enrichment analysis. Gene Set Enrichment Analysis was performed using preranked differentially expressed genes, ranked by *t* score, using the Gene Set Enrichment Analysis Preranked Gene Pattern module. The hallmark gene sets from the following link were used: https://www.gsea‐msigdb.org/gsea/index.jsp.

### Multiple Reaction Monitoring Analysis

Multiple reaction monitoring (MRM) assays were developed to quantitatively validate target proteins. Samples were digested as described, and data were normalized by the addition of 50 fmol of β‐galactosidase. MRM analysis was conducted using a QTRAP 6500 mass spectrometer (SCIEX, Framingham, MA) equipped with an LC–20AD nano‐HPLC system (Shimadzu, Kyoto, Japan). The mobile phase comprised solvent A (0.1% formic acid aqueous solution) and solvent B (98% acetonitrile plus 0.1% formic acid). Peptides were separated on a C18 column (15 cm long, 75 μm diameter, 3.6 μm beads) at a flow rate of 300 nL/min, following a gradient elution of 5% to 30% solvent B for 38 minutes, 30% to 80% solvent B for 4 minutes, and maintained at 80% for 8 minutes.

For the QTRAP 6500 mass spectrometer, a spray voltage of 2400 V, 23 psi of nebulized gas, and a dwell time of 10 milliseconds were used. Multiple MRM transitions were monitored in Q1 and Q3 quadrupoles using unit resolution to maximize specificity. A spectral library of MS/MS data was generated on an TripleTOF6500 (AB SCIEX, Foster City, CA) and searched using Mascot version 2.3 (Matrix Science, UK) against a *Homo sapiens* database (20 307 entries). MRM data were analyzed with Skyline software.

### Statistical Analysis

Comparisons of clinical characteristics between patients with recurrent and nonrecurrent AIS were performed using the Student *t* test or nonparametric Wilcoxon rank sum test for numeric variables, and the Fisher exact test for categorical variables. Statistical analyses were performed using R (version 3.2.4). Continuous data and ranked data are presented as mean±SD and median with interquartile range, respectively. Candidate markers were selected based on *P*<0.05 results from *t* tests in liquid chromatography tandem mass spectrometry analysis. MSstats with the linear mixed‐effects model were used to calculate fold change from the peptide intensity in MRM analysis. Multiple testing errors were handled by Bonferroni correction, and a false discovery rate <0.05 was considered statistically significant. Receiver operating characteristic screening was performed on the selected biomarker panels via the website https://www.metaboanalyst.ca/MetaboAnalyst/. Prism GraphPad (version 8.2.1) was used for generation of receiver operating characteristic curves as well as their 95% CIs (Wilson‐Brown method).

## Results

### Patient Characteristics

Despite adhering to guideline‐based secondary prevention, patients in the CNSR‐III cohort still face a risk of recurrence of nearly 6.7% within the initial 3 months following AIS.^15^ In this study, plasma proteins were analyzed from 214 age‐ and sex‐matched patients without diabetes or heart disease in the CNSR‐III cohort to identify potential predictive biomarkers of recurrent stroke. The subcohort consisted of 107 patients in the nonrecurrence group who experienced a single ischemic stroke and 107 patients in the recurrence group who had a recurrence within 3 months following the initial attack. Out of the total 214 patients with ischemic stroke included in the study, 192 plasma samples qualified for further analysis following mass spectrometry detection. Among the remaining 192 patients, 6 were classified as cardioaortic embolism (CCS2) and 3 as other uncommon causes (CCS4). Due to the limited number of cases with CCS2 and CCS4 for further analysis, our study primarily focused on 183 patients categorized into 1 of 3 major ischemic stroke categories based on CCS criteria: CCS1, CCS3, and CCS5. The demographic characteristics, baseline risk factors, and disability classifications for the study population are summarized in the Table.

**Table 1:** Clinical Characteristics for Different CCS Subtypes and in Combination

Characteristic	Large‐artery atherosclerosis (CCS1)		*P* value	Small‐artery occlusion (CCS3)		*P* value	Undetermined causes (CCS5)		*P* value	Combined		*P* value
	Nonrecurrence	Recurrence		Nonrecurrence	Recurrence		Nonrecurrence	Recurrence		Nonrecurrence	Recurrence	
	n=25	n=35		n=25	n=23		n=42	n=33		n=92	n=91	
Age, y	64.4±12.7	61.9±12.2	0.44	60.6±10.2	60.5±12.0	0.98	61.8±11.1	60.5±12.5	0.62	62.2±11.3	61.0±12.1	0.5
Men (%)	76	80	0.71	64	56.5	0.6	61.9	63.6	0.88	61 (66.3)	62 (68.1)	0.79
BMI	24.8±3.0	24.0±3.4	0.31	24.3±3.1	25.5±3.9	0.26	25.1±3.0	26.5±3.7	0.07	24.8±3.0	25.3±3.8	0.37
Hypertension (%)			0.55			0.56			0.55			0.83
140–159 mm Hg	8	5.9	_	20.8	17.4	_	8	5.9	_	12	8.8	_
160–179 mm Hg	8	17.6	_	20.8	34.8	_	8	17.6	_	21.7	26.4	_
≥180 mm Hg	84	76.5	_	58.3	47.8	_	84	76.5	_	64.1	62.6	_
Artery stenosis (%)	88	71.4	0.12	44	8.7	<0.01*	19	30.3	0.26	44	41.3	0.72
SBP, mm Hg	156.8±25.1	163.2±22.2	0.3	156.2±26.1	162.1±21.0	0.39	157.0±27.9	156.7±24.0	0.96	156.7±26.4	160.6±22.5	0.29
LDL, g/L	2.5 (1.9–3.4)	2.1 (1.9–3.1)	0.5	2.3 (1.7–2.7)	3.0 (1.6–3.7)	0.18	2.5 (1.8–3.1)	2.3 (1.8–3.1)	0.91	2.4 (1.8–3.0)	2.3 (1.8–3.2)	0.58
CHOL, mmol/L	4.1 (3.5–4.9)	4.0 (3.4–5.1)	0.59	3.6 (3.4–4.5)	4.8 (3.0–5.7)	0.19	3.9 (3.3–4.7)	4.0 (3.4–5.0)	0.83	3.9 (3.4–4.8)	4.0 (3.3–5.1)	0.75
hsCRP, mg/L	2.8 (0.7–11.4)	2.8 (0.9–13.0)	0.27	1.0 (0.7–3.6)	0.9 (0.6–2.6)	0.3	2.3 (0.9–6.5)	1.8 (0.8–4.7)	0.9	2.1 (0.7–6.7)	1.6 (0.7–5.7)	0.42
Homocysteine, umol/L	17.7 (13.2–25.0)	15.3 (12.1–19.7)	0.14	16.1 (12.2–18.1)	15.1 (13.8–17.5)	0.74	15.8 (12.4–20.0)	14.4 (12.1–20.1)	0.86	16.5 (12.6–20.4)	15.0 (12.3–18.9)	0.25
Thrombolysis (%)	8	14.3	0.45	16	8.7	0.44	8	14.3	0.45	10.9	9.9	0.83
NIHSS												
Admission	5.0 (2.0–8.0)	4.0 (2.0–8.0)	0.47	4.0 (2.0–7.0)	3.0 (1.0–6.0)	0.42	3.0 (1.0–5.0)	3.0 (1.0–6.0)	0.98	4.0 (2.0–6.5)	3.0 (1.0–7.0)	0.62
Discharge	3.0 (0.0–5.0)	6.0 (2.0–10.0)	0.02*	1.0 (0.0–3.0)	5.0 (1.0–9.0)	0.02*	3.0 (0.0–5.0)	6.0 (2.0–10.0)	<0.01*	2.0 (0.0–3.5)	5.0 (1.0–9.0)	<0.01*
mRS >2 (%)												
Baseline	44	45.7	0.9	40	26.1	0.31	28.6	24.2	0.67	35.9	48.4	0.97
3 mo	16	60	<0.01*	0	39.1	<0.01*	9.8	30.3	0.04*	8.7	44	<0.01*
6 mo	12	48.6	<0.01*	4.2	31.8	0.01*	12.2	27.3	0.14	9.8	36.3	<0.01*
12 mo	12.5	54.3	<0.01*	0	30.4	<0.01*	4.9	27.3	<0.01*	5.4	38.5	<0.01*

Data are presented as mean±SD, median (interquartile range), or percentage.

BMI indicates body mass index; CCS, Causative Classification of Ischemic Stroke; CHOL, total cholesterol; hsCRP, high‐sensitivity C‐reactive protein; LDL, low‐density lipoprotein; mRS, modified Rankin Scale; NIHSS, National Institutes of Health Stroke Scale; and SBP, systolic blood pressure.

* *P*<0.05.

There were no significant differences observed in the incidence of traditional risk factors for AIS, such as blood pressure, low‐density lipoprotein, and high‐sensitivity C‐reactive protein, between the recurrence and nonrecurrence groups, except for a higher rate of artery stenosis in the recurrent CCS3 subgroup compared with the nonrecurrent CCS3 subgroup at baseline. There were no significant differences noted in the degree of disability at the baseline between the 2 groups. However, after a 3‐month follow‐up, the recurrence group exhibited significantly more severe disability compared with the nonrecurrence group. These findings highlight the impact of AIS recurrence on worsening disability and emphasize the criticality of early intervention to mitigate recurrence.

### Identification of Dysregulated Plasma Proteins Based on CCS Stroke Classification

In total, liquid chromatography tandem mass spectrometry detected 1788 proteins in plasma samples from the discovery phase. Among them, we screened out 1084 proteins with call rates >30% in both the recurrence and nonrecurrence groups and analyzed them based on CCS classification. DEPs, including GNAQ (G protein subunit α Q), CA3 (carbonic anhydrase 3), ARL6IP5 (ADP ribosylation factor like GTPase 6 interacting protein 5), and SPTB (spectrin β) were identified in total participants between recurrence and nonrecurrence patients with ischemic stroke (Table S1). In addition, DBNL (Drebrin‐like protein), which regulates the innate immune system and apoptotic cleavage of cellular proteins, showed dysregulated expression across different subtypes (Figure S1, Table S2). Considering the heterogeneity of stroke cause, we further analyzed DEPs based on stroke subtypes.

In the CCS1 subgroup, the recurrence group showed downregulation of 26 proteins out of 27 DEPs compared with the nonrecurrence group. Noteworthy DEPs included the proapoptotic protein BID (BH3‐interacting domain death agonist) and TREML1 (Trem‐like transcript 1 protein), known for their involvement in neuronal cell death and platelet aggregation, respectively.^16^, ^17^

In the CCS3 subgroup, the recurrence group exhibited upregulation of 103 proteins and downregulation of 17 proteins compared with the nonrecurrence group. Among them, KAD1 (adenylate kinase isoenzyme 1) and ENO1 (α‐enolase) were identified and are known to contribute to vascular inflammation and cerebroprotection against ischemic stroke.^18^, ^19^

**Figure 2:**
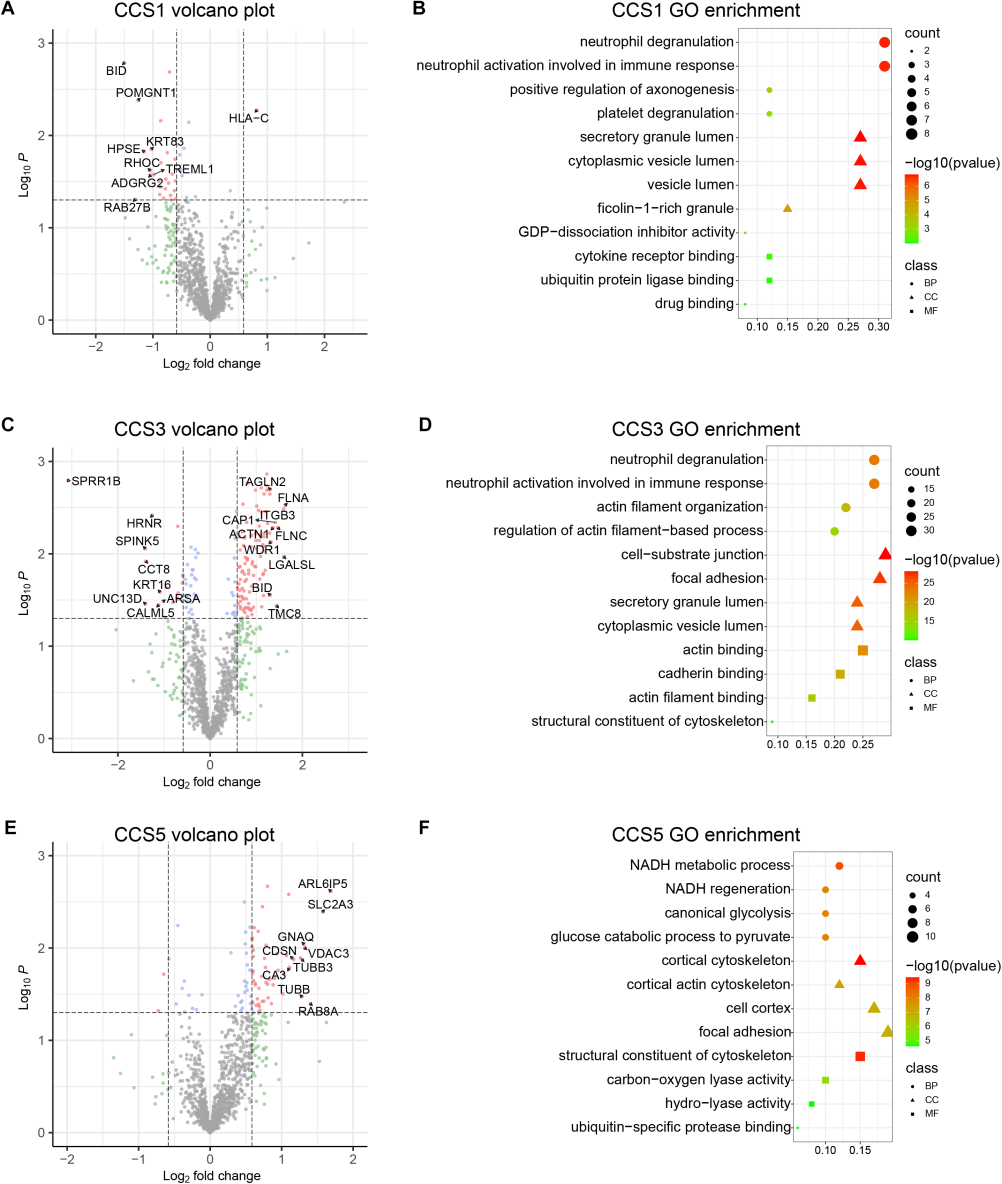
Alteration of the plasma proteome in patients with acute ischemic stroke and recurrence within 3 months. **A**, **C**, **E,** Volcano plots were generated to visualize the proteomics data comparing the recurrence and nonrecurrence groups, stratified by CCS subtype (CCS1, CCS3, and CCS5). Each dot represents a single protein (n=1084 proteins). *P*<0.05 and fold change >1.5 or <0.67 were considered differentially expressed genes, and the thresholds are indicated by dashed lines. Red dots: proteins with significantly different fold change. Blue dots and gray dots: proteins with fold change that was not statistically significant. **B**, **D**, **F**, GO enrichment analysis of dysregulated serum proteins in the recurrence group analyzed by CCS subgroup (CCS1, CCS3, and CCS5). Data were analyzed using the Student *t* test. ACTN1 indicates alpha‐actinin‐1; ADGRG2, adhesion G‐protein coupled receptor G2; ARL6IP5, ADP ribosylation factor like GTPase 6 interacting protein 5; ARSA, arylsulfatase A component B; BID, BH3‐interacting domain death agonist; BP, biological process; CA3, carbonic Anhydrase 3; CALML5, calmodulin‐like protein 5; CAP1, adenylyl cyclase‐associated protein 1; CC, cellular component; CCS, Causative Classification of Ischemic Stroke; CCT8, T‐complex protein 1 subunit theta; CDSN, Corneodesmosin; FLNA, Filamin‐A; FLNC, Filamin‐C; GNAQ, G protein subunit alpha Q; GO, gene ontology; HLA‐C, HLA class I histocompatibility antigen, C alpha chain; HPSE, Heparanase 50 kDa subunit; HRNR, Hornerin; ITGB3, Integrin beta‐3; KRT16, keratin, type I cytoskeletal 16; KRT83, keratin, type II cuticular Hb3; LGALSL, galectin‐related protein; MF, molecular function; NADH, reduced nicotinamide adenine diphosphate; POMGNT1, protein O‐linked‐mannose beta‐1,2‐N‐acetylglucosaminyltransferase 1; RAB27B, Ras‐related protein Rab‐27B; RAB8A, Ras‐related protein Rab‐8A; RHOC, Rho‐related GTP‐binding protein RhoC; SLC2A3, solute carrier family 2, facilitated glucose transporter member 3; SPINK5, serine protease inhibitor Kazal‐type 5; SPRR1B, Cornifin‐B; TAGLN2, Transgelin 2; TMC8, transmembrane channel‐like protein 8; TREML1, Trem‐like transcript 1 protein; TUBB3, Tubulin beta‐3 chain; UNC13D, protein unc‐13 homolog D; VDAC3, voltage‐dependent anion‐selective channel protein 3; and WDR1, WD repeat‐containing protein 1.

For the CCS5 subgroup, the recurrence group exhibited a significant increase in 51 out of 53 differentially expressed proteins. Notably, PRDX1 (peroxiredoxin‐1) and PARK7 (protein/nucleic acid deglycase DJ‐1) were among the differentially expressed proteins potentially involved in poststroke metabolic processes (Figure 2). PRDX1 has been associated with redox protective mechanisms and is found at higher levels in the ischemic score, penumbra, and circulation in patients with stroke.^20^ On the other hand, PARK7 serves as a biomarker for brain damage.^21^

### Functional Annotation and Biological Interpretations of Dysregulated Proteins

DEPs in the CCS1 recurrence group are involved in biological processes such as neutrophil degranulation and activation, as well as platelet degranulation through biological adhesion (such as GSTP1 [glutathione S‐transferase pi 1], TREML1). Similarly, the dysregulated proteins in the CCS3 recurrence group participate in neutrophil degranulation/activation, platelet aggregation, and actin filament‐related processes through actin filament binding (such as HRNR [hornerin]). In the CCS5 recurrence group, dysregulated proteins such as PRDX1 and CA3 are primarily associated with metabolic processes, including reduced nicotinamide adenine diphosphate (NADH), carbon–oxygen, as well as hydrolyase activity (Figure 2).

**Figure 3:**
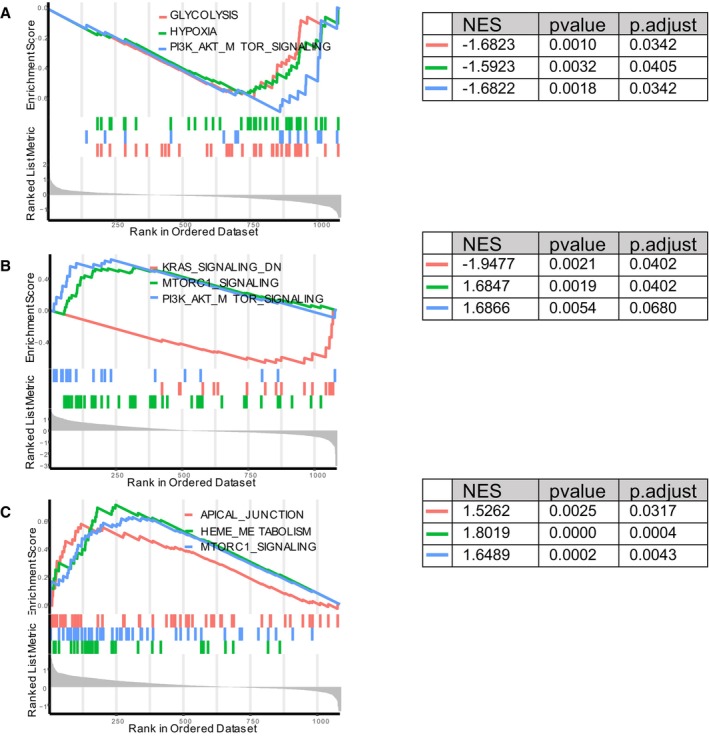
GSEA analysis based on hallmark gene sets in different CCS subtypes. **A**, Decreased expression of genes involved in regulation of glycolysis, hypoxia, and PI3K‐AKT‐MTOR signaling pathways are associated with CCS1 recurrence. **B**, Decreased expression of genes associated with KRAS signaling pathway, and increased expression of genes associated with MTORC1 signaling and PI3K‐AKT‐MTOR signaling are related to CCS3 recurrence. **C**, Increased expression of genes related to apical junction, heme metabolism, and MTROC1 signaling are associated with CCS5 recurrence. CCS indicates Causative Classification of Ischemic Stroke; GSEA, gene set enrichment analysis; KRAS, kirsten rats arcomaviral oncogene homolog; MTROC1, mammalian target of rapamycin complex 1; NES, normalized enrichment score; and PI3K‐AKT‐MTOR, phosphoinositide 3‐kinase‐protein kinase B‐mammalian target of rapamycin (PI3K‐AKT‐mTOR) pathway.

Hallmark protein signatures based on Gene Set Enrichment Analysis revealed that proteins regulating glycolysis and hypoxia were decreased in the CCS1 recurrence group. Proteins related to the kirsten rats arcomaviral oncogene homolog (KRAS) signaling pathway (genes downregulated by KRAS activation) were decreased in the CCS3 recurrence group. Additionally, proteins related to apical junction and heme metabolism were enriched in the CCS5 recurrence group. Furthermore, phosphoinositide 3‐kinase‐protein kinase B‐mammalian target of rapamycin (PI3K‐AKT‐mTOR) pathway signaling, which is related to insulin signaling, was downregulated in CCS1 and enriched in CCS3 recurrence groups. Moreover, mammalian target of rapamycin complex 1 (MRTOC1) signaling, which regulates macromolecular anabolism, was enriched in both the CCS3 and CCS5 recurrence groups (Figure 3).

### Validation of Dysregulated Proteins to Predict Recurrence of Ischemic Stroke

**Figure 4:**
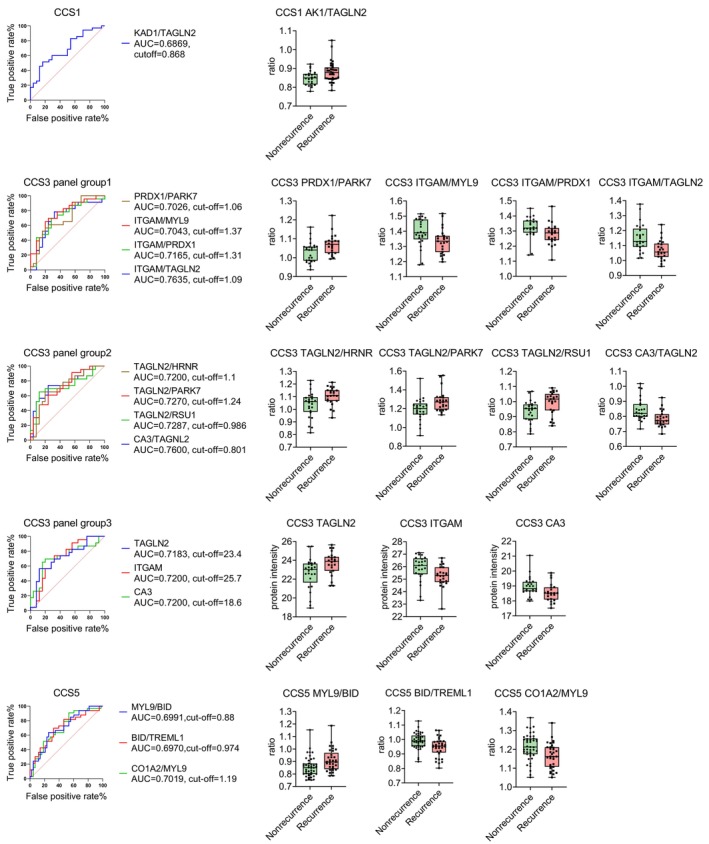
Protein panels and stroke recurrence in different CCS subtypes. **A**–**C**, Performance of candidate proteins or protein combinations in the prediction of recurrence of stroke by CCS subtypes. The left part shows the receiver operating characteristic curves of potential protein panels, and the right columns show the exact value of each biomarker in the recurrence and nonrecurrence group. AK1 indicates adenylate kinase isoenzyme 1; AUC, area under the curve; CA3, carbonic Anhydrase 3; CCS, Causative Classification of Ischemic Stroke; CO1A2, Collagen Type I Alpha 2 Chain; HRNR, Hornerin; ITGAM, Integrin Subunit Alpha M; MYL9, Myosin Light Chain 9; PARK7, protein/nucleic acid deglycase DJ‐1; PARK7, protein/nucleic acid deglycase DJ‐1; PRDX1, peroxiredoxin‐1; RSU1, Ras Suppressor Protein 1; TAGLN2, Transgelin 2; and "/", the ratio of the former protein to the latter protein.

To verify protein expressions in the recurrence group, we quantified the top 60 significantly dysregulated proteins using multiple reaction monitoring (Figure S2). Twenty of these proteins were identified in the discovery phase across different CCS subtypes. The area under the curve (AUC) was analyzed to determine the performance of these proteins to predict stroke recurrence. Protein panels were selected as potential predictors of recurrence, with an AUC value ranging from 0.69 to 0.76 (Figure 4, Table S3). The results showed that the TAGLN2 (KA1 [adenylate kinase isoenzyme 1]/transgelin 2) ratio may predict recurrence in the CCS1 subgroup (AUC=0.6869). In the CCS3 subgroup, 3 individual proteins, CA3, ITGAM (integrin subunit αM), and TAGLN2 (AUC=0.72, 0.72, and 0.7183, respectively), were predictive of recurrent stroke. Additionally, 8 protein panels had potential to predict recurrence in the CCS3 subgroup, including ITGAM/TAGLN2 ratio, CA3/TAGLN2 ratio, TAGLN2/RSU1 (Ras suppressor protein 1) ratio, TAGLN2/PARK7 ratio, TAGLN2/HRNR ratio, ITGAM/PRDX1 ratio, ITGAM/MYL9 (myosin light chain 9) ratio, and PRDX1/PARK7 ratio (AUC=0.7635, 0.76, 0.7287, 0.7270, 0.72, 0.7165, 0.7043, and 0.7026, respectively). In the CCS5 subgroup, MYL9/BID ratio, BID/TREML1 ratio, and COL1A2 (collagen type I α 2 chain)/MYL9 ratio (AUC=0.6991, 0.6970, and 0.7019, respectively) may predict recurrence. These findings suggest that individual or combinations of dysregulated proteins in plasma may identify patients with risk of recurrent stroke.

## Discussion

In this study, we conducted plasma proteomics based on the CNSR‐III cohort to investigate DEPs in patients experiencing recurrent stroke, taking into account the cause of the vascular event. The CNSR‐III cohort offers comprehensive and detailed information for patients with stroke at the current stage, and to the best of our knowledge, this research represents the largest patient population to date using plasma proteomics to assess stroke recurrence within CCS subtypes. Additionally, we quantified and verified plasma protein signatures that may help to distinguish patients at a higher risk of stroke recurrence based on the CCS subtypes. Given the comparable baseline clinical characteristics between the recurrence and nonrecurrence groups, the influence of potential confounding factors on our protein‐level study is minimized, thus providing valuable insights into the pathogenesis of stroke reccurence.^5^, ^6^, ^7^, ^8^, ^22^

In the recurrent groups of CCS1 and CCS3, the differentially expressed proteins are associated with neutrophil degranulation or activation, which may elucidate the previously observed correlations between inflammatory markers such as IL (interleukin)‐6, CRP (C‐reactive protein), and neutrophil counts and the occurrence of relapses.^23^, ^24^, ^25^, ^26^ The plasma protein environment plays a crucial role in modulating the function of neutrophils.^27^ Following a stroke event, inflammation is triggered, leading to a peripheral immune response and the subsequent systemic inflammatory response that may exacerbate chronic brain inflammation.^28^, ^29^

Additionally, the recurrent‐associated proteins in CCS1 and CCS3 are involved in platelet degranulation and aggregation, which could explain the associations between high platelet reactivity, platelet volume, D‐dimer, and recurrence.^30^, ^31^ Furthermore, the DEPs in the recurrent group CCS5 are related to NADH metabolism. The Gene Set Enrichment Analysis suggests that CCS1, CCS3, and CCS5 are associated with glucose, insulin, and hypoxia metabolism, offering some insights into the previously observed correlations between metabolic markers such as stress‐induced hyperglycemia and recurrence.^32^

Several proteins in our biomarker panels have been implicated in the pathogenesis of stroke. For instance, previous research has demonstrated that ITGAM could potentially serve as a diagnostic and prognostic biomarker, as well as a therapeutic target for unstable atherosclerotic plaque‐related stroke.^33^ Additionally, the continuous basal hyperactivation of circulating neutrophils, associated with elevated levels of ITGAM, has been linked to blood–brain barrier breakdown and increased clinical severity.^29^ To further validate these dysregulated proteins, the inclusion of independent cohorts with larger sample sizes in future studies would enhance the robustness of these findings.

In summary, in this study, we analyzed plasma proteomics in a multicenter cohort of stroke patients to identify protein markers associated with early stroke recurrence. We discovered novel protein signatures that can be measured during the acute stage of stroke to predict recurrence within 3 months based on the CCS classification. These potential prognostic biomarkers may improve medical management and risk stratification for patients with stroke. Further validation in larger prospective cohorts is needed to confirm their predictive capacity. Our findings provide valuable insights for developing targeted therapies to prevent stroke recurrence and improve poststroke outcomes.

## Sources of Funding

This work was supported in part by grants from the National Natural Science Foundation of China (82320108007, 82122021, 82001243).

## Disclosures

None.

## Supporting information


Tables S1–S3
Figures S1–S2
