# Supplementary material for: Potential Protein Signatures for Recurrence Prediction of Ischemic Stroke
Source: J Am Heart Assoc. 2024 Feb 29;13(5):e032840. doi: 10.1161/JAHA.123.032840 (PMC10944055; doi:10.1161/JAHA.123.032840)
Supplement: Supplementary file 1 — Tables S1–S3 Figures S1–S2 [file JAH3-13-e032840-s001.pdf]

SUPPLEMENTAL MATERIAL

Table S1. Differentially expressed proteins at baseline in total.

Protein	Fold_change	Pvalue
GNAQ	1.528	0.05
CA3	1.503	0.03389
ARL6IP5	1.724	0.025
SPTB	1.572	0.003219

Table S2. Examples of differentially expressed proteins at baseline by CCS subtype.

Protein	Full_name	CCS1		CCS3		CCS5	
		Fold change	*P* value	Fold change	*P* value	Fold change	*P* value
TAGLN2	Transgelin-2	0.77	0.29	2.48	**0.00***	1.57	0.07
BID	BH3-interacting domain death agonist	0.35	**0.00***	2.47	**0.03***	1.27	0.41
ZYX	Zyxin	0.88	0.69	2.21	**0.02***	1.87	**0.02***
RSU1	Ras suppressor protein 1	0.78	0.37	1.86	**0.04***	1.61	**0.04***
ENO1	Alpha-enolase	0.73	0.15	1.69	**0.02***	1.55	**0.02***
TREML1	Trem-like transcript 1 protein	0.51	**0.03***	1.57	**0.02***	1.24	0.33
PGAM1	Phosphoglycerate mutase 1	0.73	0.10	1.57	**0.01***	1.53	**0.01***
GSTO1	Glutathione S-transferase omega-1	0.83	0.27	1.49	**0.00***	1.41	**0.01***
AK1	Adenylate kinase isoenzyme 1	0.95	0.67	1.42	**0.03***	1.25	0.11
CCL18	C–C motif chemokine 18	1.99	0.06	1.29	0.23	1.27	0.34
PARK7	Protein/nucleic acid deglycase DJ-1	0.91	0.68	1.28	0.26	1.72	**0.01***
ERAP2	Endoplasmic reticulum aminopeptidase 2	0.77	0.31	1.26	0.43	0.96	0.90
MYL9	Myosin regulatory light polypeptide 9	1.84	0.15	1.19	0.49	2.13	0.06
CA3	Carbonic anhydrase 3	1.15	0.68	1.18	0.64	2.16	**0.02***
SAA1	Serum amyloid A-1 protein	1.18	0.73	1.15	0.71	1.06	0.86
PRDX1	Peroxiredoxin-1	0.92	0.52	1.14	0.39	1.50	**0.01***
ITGAM	Integrin alpha-M	3.31	0.15	0.73	0.68	1.95	0.27
HRNR	Hornerin	0.84	0.52	0.42	**0.00***	1.47	0.06

Table S3. AUC value and 95% confidence interval for each panel for CCS subtypes.

CCS subtypes	Panel	AUC	95% CI	P value
CCS1	AK1/TAGLN2	0.6869	0.5505 — 0.8233	0.0154
CCS3	PRDX1/PARK7	0.7026	0.5537 — 0.8515	0.0162
CCS3	ITGAM/MYL9	0.7043	0.5496 — 0.8591	0.0153
CCS3	ITGAM/PRDX1	0.7165	0.5677 — 0.8653	0.0102
CCS3	ITGAM/TAGLN2	0.7635	0.6282 — 0.8988	0.0018
CCS3	TAGLN2/HRNR	0.72	0.5723 — 0.8677	0.009
CCS3	TAGLN2/PARK7	0.727	0.5849 — 0.8690	0.0071
CCS3	TAGLN2/RSU1	0.7287	0.5781 — 0.8793	0.0067
CCS3	CA3/TAGLN2	0.76	0.6210 — 0.8990	0.002
CCS3	TAGNL2	0.7183	0.5704 — 0.8662	0.0096
CCS3	ITGAM	0.72	0.5734 — 0.8666	0.009
CCS3	CA3	0.72	0.5691 — 0.8709	0.009
CCS5	MYL9/BID	0.6991	0.5816 — 0.8165	0.0031
CCS5	BID/TREML1	0.697	0.5765 — 0.8174	0.0034
CCS5	CO1A2/MYL9	0.7019	0.5841 — 0.8197	0.0027

Figure S1. Venn diagram of differentially expressed proteins detected by LC-MS.

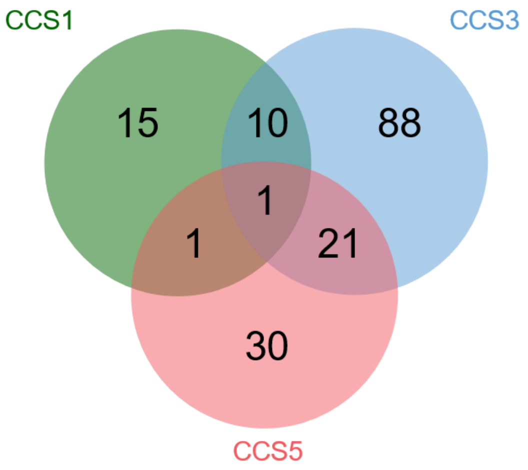

**Common DEG**	
CCS1 CCS3	BID、TREML1、TPM3、FERMT3、GSTP1、AK2、GPI、ARHGDIA、GDI1
CCS1 CCS5	SH3BGRL3
CCS3 CCS5	HSPA1A,HSPA1B、PNP、PGAM1、CAPN1、TPI1、SH3BGRL、ENO1、VCP、MTPN、PRDX5、TPM4、NAP1L1、RSU1、TUBA1B、GAPDH、CFL1、YWHAZ、ZYX、ACTB、WDR1、CAP1
CCS1 CCS3 CCS5	DBNL

Figure S2. MRM development outline.

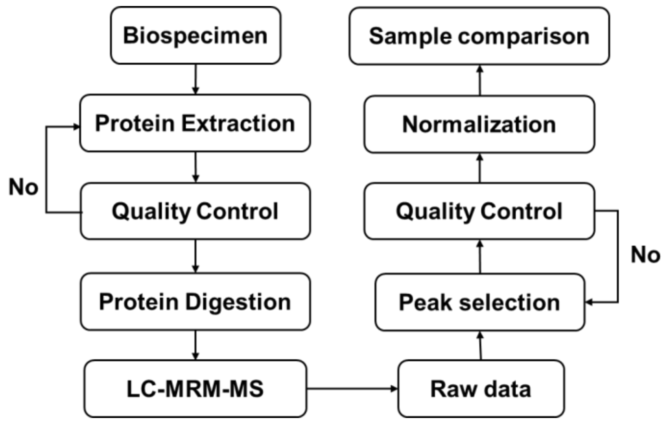

The final MRM method included 60 proteins selected from the discovery cohort.
